# PRRDB 2.0: a comprehensive database of pattern-recognition receptors and their ligands

**DOI:** 10.1093/database/baz076

**Published:** 2019-06-27

**Authors:** Dilraj Kaur, Sumeet Patiyal, Neelam Sharma, Salman Sadullah Usmani, Gajendra P S Raghava

**Affiliations:** 1 Department of Computational Biology, Indraprastha Institute of Information Technology, Delhi, New Delhi 110020, India; 2 Bioinformatics Centre, CSIR-Institute of Microbial Technology, Chandigarh, Chandigarh 160036, India

## Abstract

PRRDB 2.0 is an updated version of PRRDB that maintains comprehensive information about pattern-recognition receptors (PRRs) and their ligands. The current version of the database has ~2700 entries, which are nearly five times of the previous version. It contains extensive information about 467 unique PRRs and 827 pathogens-associated molecular patterns (PAMPs), manually extracted from ~600 research articles. It possesses information about PRRs and PAMPs that has been extracted manually from research articles and public databases. Each entry provides comprehensive details about PRRs and PAMPs that includes their name, sequence, origin, source, type, etc. We have provided internal and external links to various databases/resources (like Swiss-Prot, PubChem) to obtain further information about PRRs and their ligands. This database also provides links to ~4500 experimentally determined structures in the protein data bank of various PRRs and their complexes. In addition, 110 PRRs with unknown structures have also been predicted, which are important in order to understand the structure–function relationship between receptors and their ligands. Numerous web-based tools have been integrated into PRRDB 2.0 to facilitate users to perform different tasks like (i) extensive searching of the database; (ii) browsing or categorization of data based on receptors, ligands, source, etc. and (iii) similarity search using BLAST and Smith–Waterman algorithm.

## Introduction

Innate immunity is ubiquitous, naturally present in all classes of plants and animals, and acts as the first line of defense. Innate has originated from the Latin word ‘Innatus’, which stands for ‘Inborn’. It comprises cells and mechanism providing the first line of defense against invading pathogens in a nonspecific manner. Besides macrophages and dendritic cells, nonprofessional cells such as fibroblasts and endothelial and epithelial cells play a significant role in pathogen recognition (1). Innate immunity also contributes to acute inflammation induced by tissue damage or microbial infection and also activates the acquired immunity. Innate immune cells utilize germline-encoded specialized receptors known as pattern recognition receptors (PRRs) to identify the pathogen- and damage-associated molecular patterns (PAMPs and DAMPs, respectively) present on invading microbes (2, 3).

**Figure 1:**
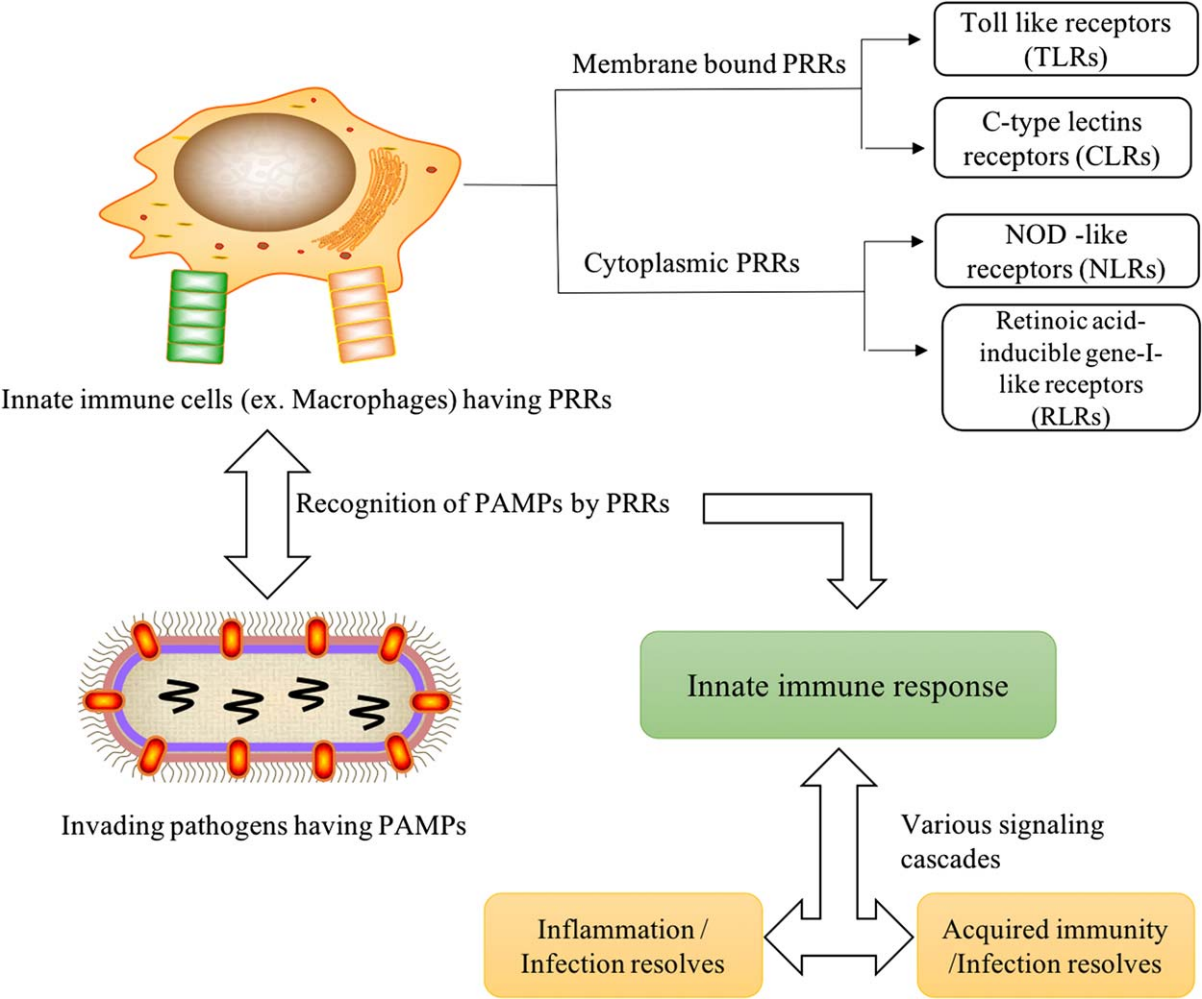
Graphical representation of immune mechanism through PRRs and PAMPs association after the microbial invasion.

In the past, different PRR families have been studied, which mainly include transmembrane proteins such as Toll-like receptors (TLRs) and C-type lectins receptors (CLRs) and cytoplasmic proteins such as nucleotide-binding oligomerization domain (NOD)-like receptors (NLRs) and retinoic acid-inducible gene-I-like receptors (RLRs). TLRs are type-1 transmembrane proteins and are the most extensively studied PRRs, which detect PAMPs associated with invading pathogens outside of the cell and in intracellular endosomes and lysosomes (4–8). CLRs are the signaling transmembrane CLRs, which play essential roles in antifungal immunity. CLRs are characterized by at least one C-type lectin-like domain and recognize both exogenous as well as endogenous ligands (6, 9). NLRs and RLRs are intracellular cytosolic sensors. NLRs, generally associated with bacterial recognition, are composed of a central nucleotide binding domain and C-terminal leucine-rich repeats, whereas RLRs are the helicases that sense PAMPs with viral RNA (10, 11). In addition to this, several other receptors such as scavenger receptors, mannose receptors and β-glucan receptors induce phagocytosis. Some secreted PRRs, such as complement receptors, collectins, ficolins, pentraxins for instances, serum amyloid and C-reactive protein, lipid transferases, peptidoglycan recognition proteins (PGRs), XA21D, etc., are not associated with their productive cells (12).

The primary function of all the PRRs is to recognize the essential microbial components, i.e. PAMPs or DAMPs. The association of PRRs with PAMPs leads to diverse phenomena such as maturation, migration and activation of immune cells, secretion of cytokines and chemokines (13). Most of the PRRs upregulate the transcription of genes regulating the proteins involved in inflammatory response such as type I interferons (IFNs), proinflammatory cytokines, chemokines, antimicrobial proteins, etc. They also upregulate the transcription and translation of proteins involved in the modulation of PRR signaling that may traverse to the adaptive immune response (2, 14, 15) (Figure 1). Adaptive immune system or ‘specific immune system’ is composed of specific cells and includes humoral and cell-mediated immunity, which aids in eliminating pathogens in the late phase of infection. Based on PRR, the innate immune system easily differentiates the type of pathogens and thus recruits the most effective form of adaptive immune response to destroy the pathogens and their toxic molecules (16–18). This field has been extensively studied in the past, and researchers have developed several computational resources like MHCBN, IEDB, Bcipep, etc. (19–21). BepiPred 2.0, Bcepred, Lbtope, IgPred, PEASE, etc. aid in predicting epitopes in humoral-mediated immunity (22–26). ProPred 1 and NetMHCstabpan help in predicting MHC-I binder, whereas ProPred, MHC2Pred and EpiDOCK predict MHC-II binders (27–32). Furthermore, methods for predicting T-helper epitopes based on cytokines, like IFN$-\gamma$, IL-4 and IL-10 (33–35), have also been explored. Recently, we have published review articles, which provide detailed insights about the immunological resources and *in silico* tools (36, 37). We believe that computational resources in the field of PRRs–PAMPs need revival. How specific PRRs sense the invading pathogens, mechanisms involved in immune response against PAMPs, downstream signaling cascades involved in eliciting immune response, etc. are some questions/challenges that must be clearly inferred to retain better therapeutic strategies against several infectious diseases caused by invading pathogens.

**Figure 2:**
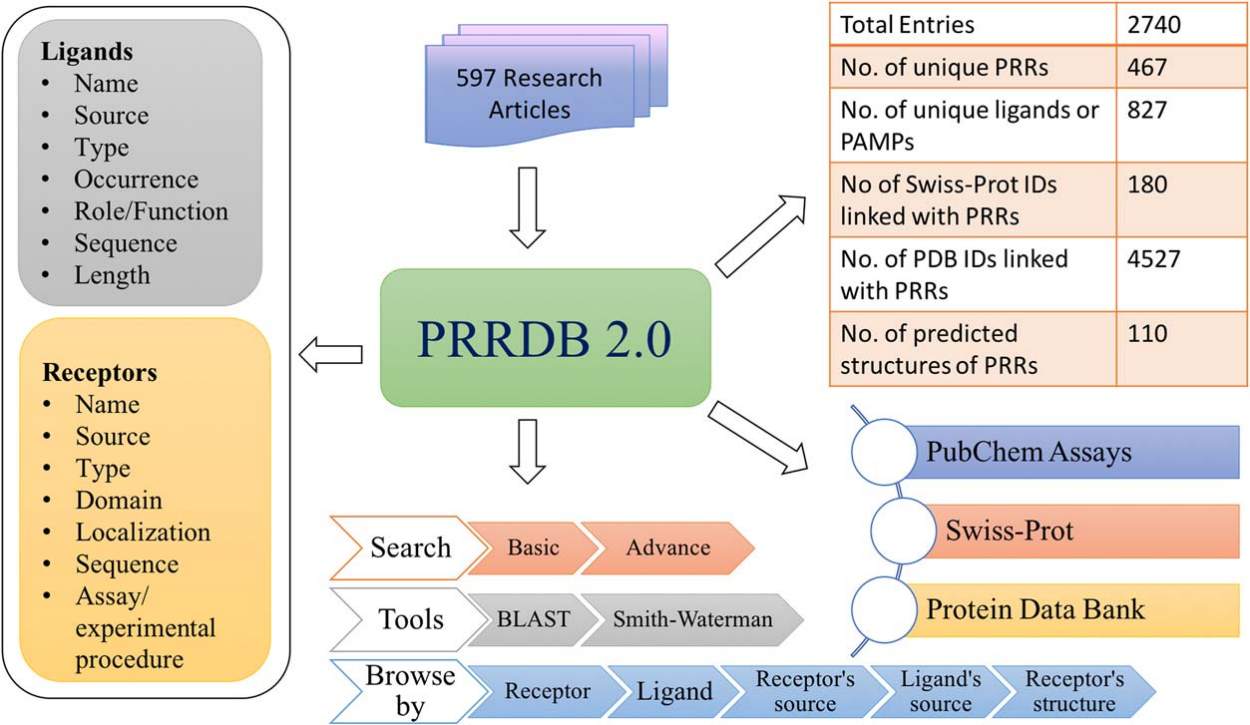
Architecture of PRRDB 2.0.

The first version of PRRDB, a comprehensive database of PRRs and their ligands, was developed in 2008 (38). PRRDB aided in developing new resources such as AntigenDB (39) and PolysacDB (40) as well as facilitated the prediction of pattern receptor recognition families (41). Since 2008, the understanding of innate immunity has been strengthened, and several other pathogen-associated molecules have been studied and discovered. Therefore, there is a necessity to enhance and upgrade the information about PRRs and their ligands in as much detail as possible. PRRDB 2.0 is an updated comprehensive resource of PRRs and their ligands. The updated version has exhaustive information about receptors such as about their domain, localization, as well as elaborative functions with role, occurrence and sequence information of their ligands. In addition to this, PubChem assays and experimental procedures, while elucidating the PRRs and their ligands as well as their available structures, have been included in the updated version, which were previously lacking.

## Material and methods

### Data collection and compilation

PubMed was mined using keywords ‘Pattern recognition receptors’ and ‘Pathogen-associated molecular patterns’, specifically published from 2008 to 2018. The cumulative hits obtained were ~30 000. We manually screened all the abstracts, and ~3000 abstracts were selected for further data mining. Final data was obtained from 597 research articles.

### Database architecture and web interface

PRRDB 2.0 was built on an Apache HTTP server (version 2.4.7) installed on a machine with Ubuntu (version 14.04.5) as the operating system. MySQL (version 5.5.55) was used as the back end to manage the data, while HTML5, PHP5, CSS3 and JAVA scripts were used for developing responsive front ends, compatible for mobiles, tablets and desktops. PERL and PHP programming language were used to develop a common interface. The architecture of PRRDB 2.0 is depicted in Figure 2.

### Data content

Primary data contains the sole information extracted from the corresponding research articles, which has been linked under head ‘PMID’. Information has been provided in the tabular form for both ligands as well as PRRs. The main fields illustrating about ligand (PAMPs/DAMPs) are (i) Ligand name: represents the name of ligands used in the literature; (ii) Ligand source: describes the actual source or origin of that ligand; (iii) Ligand type: represents the category of ligands such as lipid, peptidoglycan, lipopolysaccharide, protein etc.; (iv) Occurrence of ligand: represents either natural or synthetic occurrence of ligands; (v) Role of ligands: provides extensive information about corresponding ligands’ role in activating the immune system. Besides this information, PRRs have been curated under heads such as (vi) Receptor’s name: represents the name of PRRs used in the literature; (vii) Receptor source: describes the actual source or origin of that receptor; (viii) Receptor type: represents the major classification of PRRs such as TLRs, CLRs, RLRs, NLRs, etc.; (ix) Localization of receptor: conveys its localization or cell type from which it has been found; (x) Domain: represents the specific domain present within particular PRRs such as Leucine-rich domain in TLR9, lectin domain in CLRs, etc. and (xi) Function: describes the role or function of PRRs in eliciting the innate immune system by signal cascades, when associated with their specific ligands. In addition to this, the experimental procedure or specific assays used in the corresponding literature is also curated under the head ‘Assay used’. As a supplement to above information, all the PubChem assays known till date, of that particular PRR, have also been provided by a hyperlink (42). PubChem and Swiss-Prot were mined to curate several important information that was not mentioned in that particular research article, such as sequences of PRRs, as well as their ligands (43). Protein Data Bank (PDB) was also queried to extract the experimentally known structures of PRRs (44). In cases where the structures were not experimentally characterized, we tried to provide the predicted structures of PRRs by virtue of structure prediction methods mainly by PHYRE2 (45).

### Implementation of tools

#### Data search

Basic and advanced search modules have been implemented in PRRDB 2.0 to provide effortless searching. The user can query against the name, source and type of ligand. Similarly, queries can also be put against name, type, source and domain of receptors. The basic search module allows the output customized according to the search query, while the user can give multiple queries simultaneously with Boolean expressions (e.g. AND, OR and NOT) in the advanced search module.

#### Data browse

Data can be browsed by (i) receptors, (ii) ligands, (iii) receptor’s source and (iv) ligand’s source. Apart from this, the receptor’s structure is an additional service to provide extensive structural information of all PRRs known till date. A user can browse all the PRRs by major type of PRRs, notably: (i) CLRs, (ii) Gram-negative binding proteins, (iii) 1,3-β-D-Glucan pattern recognition protein (GRP), (iv) Lectins, (v) Mannose receptors, (vi) NLRs, (vii) Peptidoglycan recognition proteins, (viii) Rig-like receptors, (ix) Scavenger receptors, (x) TLRs and few others. Browsing by receptor’s structure leads to a list of the particular type of PRRs and their all experimentally validated structures with PDBID, description and the source organism.

#### Data similarity or alignment

BLAST (46) and Smith–Waterman algorithm (47) have been implemented to facilitate data similarity-based search. In BLAST module, the user has to submit FASTA format of protein ligands or PAMPs/DAMPs with default or chosen parameters, and the server automatically performs the BLAST search against stored data. Similarly, Smith–Waterman algorithm also performs similarity-based search against ligands.

## Results

### Data analysis

The updated version of PRR database, PRRDB 2.0, holds a total of 2740 entries extracted from 597 research articles. We have performed a major update on PRR and their ligands. We have incorporated 2374 new receptors in addition to 353 receptors from the first version of PRRDB, making the total tally of 2727 total PRRs. Similarly, PRRDB 2.0 contains 2197 total ligands studied in the last 10 years as well as 353 ligands from the previous database, making the total count of ligands to 2550. In summary, PRRDB 2.0 comprises 2740 entries providing information about 2727 total, 467 unique PRRs and 2550 total and 827 unique ligands.

**Figure 3:**
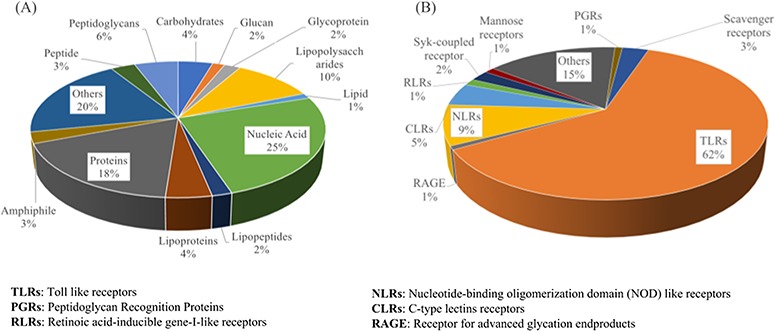
Distribution of various categories of PRRs (**A**) and ligands (**B**) stored in PRRDB 2.0.

Figure 3A shows the major type of receptors available in PRRDB 2.0. TLRs are the most prominent and widely studied among PRRs, so 62% of entries provide information regarding them. Other entries provide information about NLRs—241, CLRs—135, Scavenger—88, Syk-coupled CLRs—63, RLRs—40, Mannose receptor—33, PGRPs—25 and RAGE—22.

**Figure 4:**
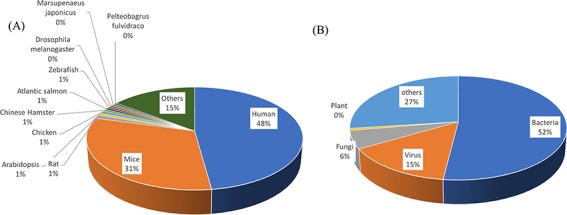
Distribution of source of PRRs (**A**) and ligands (**B**).

It is a well-known fact that TLRs and CLRs are membrane-bound pathogen receptors and around 72% of the PRRs curated in PRRDB 2.0 fall in this membrane-bound category as compared to 10% of the cytoplasmic PRRs. Similarly, Figure 3B represents the graphical distribution of entries of different ligand types that include 496 entries about nucleic acids, 353 for protein-type ligands, as well as lipopolysaccharides—207, peptidoglycan—111, carbohydrates—88, lipoproteins—85, glycoprotein—41, lipopeptide—37, glucan—31, lipid—25, polysaccharide—16, amphiphile—53 and few others. Most of the ligands (79%) stored in PRRDB 2.0 have natural origins. Data analysis also revealed that sources of most of the PRRs are human (48%) and mice (31%). These PRRs bind to ligands originated mostly from bacteria (52%), viruses (15%) and fungi (6%) (Figure 4).

### Comparison with the previous version

PRRDB was developed in 2008 and comprises two different tables; one of PRRs containing 491 entries provides information such as receptor’s name, source organism, sequence and their length, family and type. Another table is of ligands containing 266 entries, providing information like name, source, class of the ligand, origin and its receptor (38). In the updated version, we have incorporated more information about each PRR as well as ligand, so the final data consist of 2740 entries. We have tried to provide the role of ligand in activating the immune system in addition to its name, source, type and origin. Similarly, the updated version contains extensive information about each PRR such as its name, source, type, sequence and its length, localization and domain as well as its function. A comparative statistics is shown in Table 1. It shows only the major update of the database. The comprehensive statistics have been given in Supplementary Table S1. The experimental procedure or assay is also curated in the updated version. To add more to this, data has been linked with Swiss-Prot, PubChem and PDB for maximum information.

**Table 1:** Comparison of data in two versions of PRRDB database, total entries/information in PRRDB and PRRDB 2.0 is shown

**Overall information**			**Entries for different types of receptors**		
**Field/Information**	**PRRDB**	**PRRDB 2.0**	**Receptors**	**PRRDB**	**PRRDB 2.0**
Total receptors	353	2727	TLR^*^	185	1737
Total ligands	354	2550	CLR^*^	27	135
Sequence of Receptors	221	1784	NLR^*^	15	241
Sequence of ligands	241	1583	Mannose	26	33
			Scavenger	53	88
**Entries for major types of ligands**			**Entries for major sources of receptors**		
**Field/Information**	**PRRDB**	**PRRDB 2.0**	**Receptors**	**PRRDB**	**PRRDB 2.0**
Peptide	15	62	Human	146	1092
Nucleic acid	68	496	Mice	102	717
PAMP^*^	54	376	Chicken	0	17
DAMP^*^	0	247	Hamster	15	16
Protein	60	353	Rat	3	27
LPS^*^	16	207	Zebrafish	0	13
Peptidoglycan	8	111	*Arabidopsis*	1	17
Carbohydrates	37	88			

^*^TLR: Toll-like receptors, CLR: C-type lectin receptor, NLR: nucleotide-binding oligomerization domain (NOD)-like receptor, PAMP: pathogens-associated molecular pattern, DAMP: damage-associated molecular pattern, LPS: lipopolysaccharide.

### Utility of database

**Figure 5:**
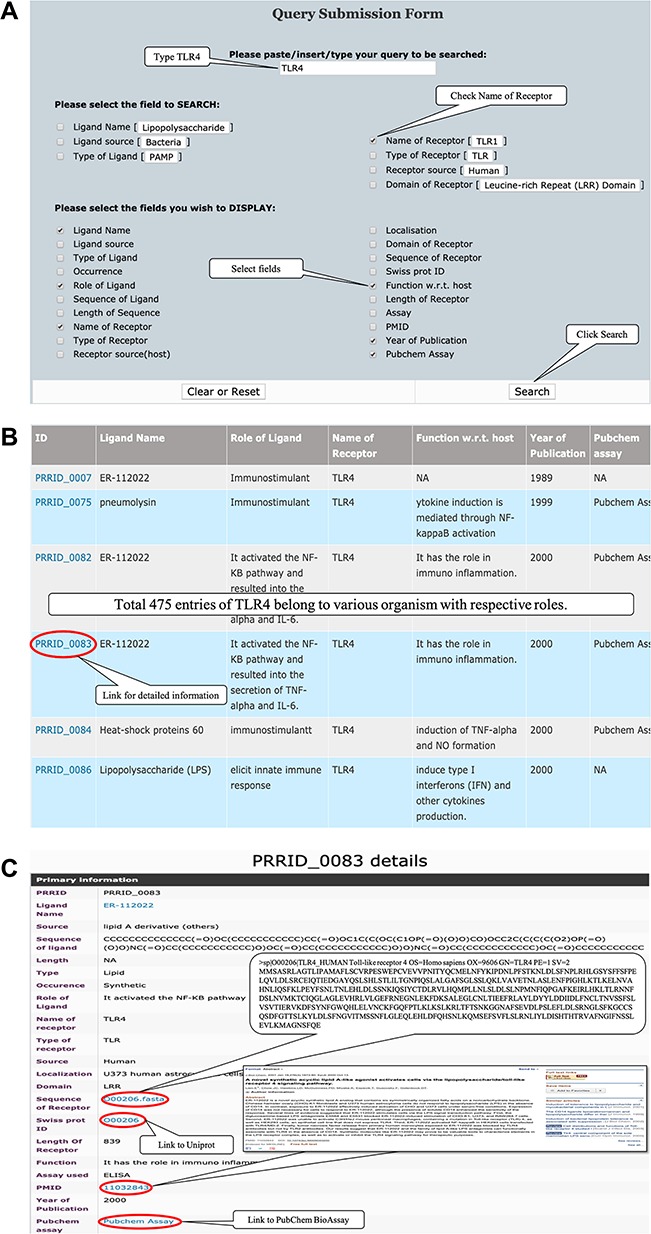
(**A**) Representative screenshot of PRRDB 2.0 demonstrating the submission of a query in simple search page. (**B**) Screenshot of PRRDB 2.0 demonstrating the result page after submission of a query in simple search page. (**C**) Screenshot of PRRDB 2.0 demonstrating the detailed information page after the result page.

PRRDB 2.0 can be used to get exhaustive information about any PRR on a single platform. For example, if a user is interested in TLR 4, which mostly recognizes lipopolysaccharides associated with gram-negative bacteria, one has to type TLR4 in the search box given at basic search page and check the name of receptor, as shown in Figure 5A. By a simple click on the search button, one will be directed towards a list of 475 entries stored in PRRDB 2.0, which are differentiated with a unique ID, as shown in Figure 5B. A click on each ID will direct to a detailed display page, providing all the information as well as hyperlinks to PubChem, PubMed and Swiss-Prot. In addition, sequence of TLR4 is also available in FASTA format as depicted in Figure 5C. Furthermore, all 27 experimentally proven structures about TLR4 and its complexes stored in PDB can also be viewed by user in PRRDB 2.0.

### Limitation and update

We have tried to provide the complete information of PRRs and their ligands, but sequences of some PRRs and ligands are neither available in the literature nor in any other chemical database. The data is manually curated and thoroughly checked to minimize the error, but claiming perfect accuracy would be unfair due to human errors that may have crept in. The first version was released 10 years ago, but we will try to update the database frequently, preferably after 3 years.

## Discussion

Innate immune system acts as the first line of defense for host and is responsible for pathogen recognition and elicitation of proinflammatory responses against invading pathogens in the initial phases of infection, whereas, in the late phase, adaptive immune system eliminates the pathogen and generates immunological memory. As elaborated in the introduction, the repertoire of PRRs is very vast and recognizes various classes of pathogens. A surprising aspect is the similar mechanism of host PRRs in recognizing a wide range of microbes in different life cycles and with different biochemical compositions. Another astonishing fact is that all classes of pathogens are sensed by more than one type of PRRs through various ligands and lead to a rapid proinflammatory response through various intracellular signal cascades (14).

Inflammation plays an important role in various infectious diseases and autoimmunity; therefore, targeting signal cascades and process of activation of the innate immune system holds a tremendous medical promise (48, 49). The concept of pattern recognition by a large family of receptors redefined the specificity and complexity of innate immune system, which was largely ignored in the early immunological studies, but emerged as a hotspot in medical interventions. Immunotherapy and vaccine have always proved to be a rescue for mankind; as a result of which, several deadly diseases have been eradicated in the past (50). Various computational tools have aided in immunotherapy and vaccine designing; a few studies have also focused on innate immune system to design therapeutics (33, 51–55). In the last decade, several databases and *in silico* tools related to therapeutic entities have been developed (56–62) and successfully used in designing newer therapeutic entities (63, 64).

Despite immense progress in innate immune-related research in the last few decades, uncertainty still exists. TLRs are the most studied PRRs, but cytoplasmic PRRs also play a major role in accumulating various immune responses, which needs more investigation. Similarly, other PRRs such as mannose receptors, scavenger receptors, as well as few secreted PRRs need to be explored further. Understanding of cross-talk between various PRRs needs better insight. We believe that PRRDB 2.0 will aid in getting all the information and queries discovered in the past.

## Conclusion

PRRDB 2.0, comprising more than 2700 entries, provides better coverage of all the PRRs and their ligands studied till now. We have improved the data coverage by incorporating additional fields and also highlighting the role and specificity of PRRs and ligands in eliciting the immune response. The hyper linkage with Swiss-Prot, PDB and PubChem will provide maximum information at a single place. We believe that the updated version will be very helpful to the scientific community.

### Availability

PRRDB 2.0 is freely available at https://webs.iiitd.edu.in/raghava/prrdb2/ as a user-friendly, display compatible interface. The previous version can be accessed at http://crdd.osdd.net/raghava/prrdb/.

## Statement of Ethics

The authors have no ethical conflicts to disclose.

## Author contributions

D.K., S.P. and N.S. manually collected and curated all the data, as well as experimentally verified structures. D.K. predicted the structures. D.K., S.P., N.S. and S.S.U. developed the web interface. S.S.U. and G.P.S.R. prepared the manuscript. G.P.S.R. conceived the idea, planned and coordinated the entire project.

## Supplementary Material

supplementary_S1_baz076

## Notes

**Database URL:** https://webs.iiitd.edu.in/raghava/prrdb2/
