# Supplementary material for: PRRDB 2.0: a comprehensive database of pattern-recognition receptors and their ligands
Source: Database (Oxford). 2019 Jun 27;2019:baz076. doi: 10.1093/database/baz076 (PMC6597477; doi:10.1093/database/baz076)
Supplement: supplementary_S1_baz076 [file supplementary_s1_baz076.docx]

**Supplementary File S1: Complete statistics of PRRDB2.0**

***Note** : PRRDB 2.0 = PRRDB(old) + PRRDB(new)

**Table 1** Detailed comparison between older and updated version of PRRDB.

|  | **PRRDB(old)** | **PRRDB(new)** | **PRRDB 2.0** |
| --- | --- | --- | --- |
| Unique papers covered | 175 | 422 | 597 |
| Total receptors | 353 | 2374 | 2727 |
| Unique receptors | 70 | 422 | 467 |
| Unique type of receptors | 16 | 41 | 50 |
| Total ligands | 354 | 2196 | 2550 |
| Unique ligands | 181 | 697 | 827 |
| Unique Type of ligands | 28 | 60 | 67 |
| Total sequences of receptors found | 327 | 1457 | 1784 |
| Unique receptor’s sequences | 44 | 140 | 180 |
| Total ligand’s sequence | 244 | 1339 | 1583 |
| Unique ligand’s sequence | 85 | 231 | 270 |

**Table 2** Detailed comparison between older and updated version of PRRDB in terms of receptors present.

|  | **PRRDB(old)** | **PRRDB(old)** | **PRRDB(new)** | **PRRDB(new)** | **PRRDB2.0** | **PRRDB2.0** |
| --- | --- | --- | --- | --- | --- | --- |
| Receptors | Total entries | Unique entries | Total entries | Unique entries | Total entries | Unique entries |
| TLR* | 185 | 10 | 1552 | 17 | 1737 | 17 |
| CLR* | 27 | 7 | 108 | 10 | 135 | 10 |
| Mannose receptor | 21 | 4 | 11 | 6 | 32 | 2 |
| Scavenger receptor | 53 | 8 | 35 | 8 | 88 | 8 |
| NLR* | 15 | 2 | 226 | 15 | 241 | 15 |
| RLR* | 5 | 2 | 35 | 2 | 40 | 2 |
| PGRPs* | 0 | 0 | 25 | 7 | 25 | 7 |
| RAGE* | 1 | 1 | 21 | 2 | 22 | 2 |
| Syk coupled CLR | 0 | 0 | 63 | 2 | 63 | 2 |
| Others | 47 | 15 | 295 | 176 | 438 | 37 |

* TLR: Toll like receptors, CLR: C-type lectins receptors, NLR: Nucleotide-binding oligomerization domain (NOD) like receptors, RLR: Retinoic acid-inducible gene-I-like receptors, PGRPs: Peptidoglycan Recognition Proteins, RAGE: Receptor for advanced glycation end products

**Table 3** Detailed comparison between older and updated version of PRRDB in terms of ligands present**.**

|  | **PRRDB(old)** | **PRRDB(old)** | **PRRDB(new)** | **PRRDB(new)** | **PRRDB2.0** | **PRRDB2.0** |
| --- | --- | --- | --- | --- | --- | --- |
| Ligands | Total entries | Unique entries | Total entries | Unique entries | Total entries | Unique entries |
| Peptide | 15 | 14 | 47 | 25 | 62 | 35 |
| Nucleic acid | 68 | 32 | 428 | 120 | 496 | 139 |
| PAMP* | 54 | 15 | 322 | 170 | 376 | 170 |
| DAMP* | 0 | 0 | 247 | 45 | 247 | 45 |
| Protein | 60 | 29 | 293 | 104 | 353 | 128 |
| LPS* | 16 | 1 | 191 | 1 | 207 | 5 |
| Peptidoglycan | 8 | 2 | 103 | 1 | 111 | 16 |
| Carbohydrates | 37 | 27 | 51 | 23 | 88 | 44 |
| Lipoproteins | 43 | 16 | 42 | 14 | 85 | 32 |
| Glycoprotein | 12 | 8 | 29 | 14 | 41 | 21 |
| lipopeptide | 6 | 3 | 31 | 13 | 37 | 14 |
| Glucan | 1 | 1 | 30 | 1 | 31 | 4 |
| Lipid | 11 | 8 | 14 | 8 | 25 | 16 |
| Polysaccharide | 5 | 1 | 11 | 1 | 16 | 1 |
| Amphiphile | 8 | 1 | 45 | 1 | 53 | 1 |
| others | 55 | 33 | 330 | 101 | 385 | 181 |

*PAMP: Pathogen associated molecular pattern, DAMP: Damage associated molecular pattern, LPS: Lipopolysaccharide.

**Table 4** Detailed comparison between older and updated version of PRRDB in terms of sources of receptors.

|  | **PRRDB(old)** | **PRRDB(old)** | **PRRDB(new)** | **PRRDB(new)** | **PRRDB2.0** | **PRRDB2.0** |
| --- | --- | --- | --- | --- | --- | --- |
| Receptor’s  source | Total entries | Unique entries | Total entries | Unique entries | Total entries | Unique entries |
| Human | 146 | 1 | 946 | 5 | 1092 | 6 |
| Mice | 55 | 8 | 662 | 20 | 717 | 25 |
| Chicken | 0 | 0 | 17 | 1 | 17 | 1 |
| Chinese Hamster | 15 | 1 | 1 | 1 | 16 | 1 |
| Atlantic salmon   (Salmo salar) | 0 | 0 | 14 | 1 | 14 | 1 |
| Drosophila Melanogaster | 3 | 1 | 9 | 1 | 12 | 1 |
| Rat | 3 | 1 | 24 | 1 | 27 | 1 |
| Zebrafish | 0 | 0 | 13 | 1 | 13 | 1 |
| Arabidopsis | 1 | 1 | 16 | 1 | 17 | 1 |
| Pelteobagrus fulvidraco | 0 | 0 | 12 | 1 | 12 | 1 |
| Marsupenaeus japonicus | 0 | 0 | 13 | 1 | 13 | 1 |
| others | 35 | 14 | 300 | 111 | 335 | 124 |

**Table 5** Detailed comparison between older and updated version of PRRDB in terms of sources of ligands.

|  | **PRRDB(old)** | **PRRDB(old)** | **PRRDB(new)** | **PRRDB(new)** | **PRRDB2.0** | **PRRDB2.0** |
| --- | --- | --- | --- | --- | --- | --- |
| Ligand’s  source | Total entries | Unique entries | Total entries | Unique entries | Total entries | Unique entries |
| Bacteria | 116 | 40 | 961 | 160 | 1077 | 177 |
| Fungi | 12 | 5 | 106 | 34 | 118 | 35 |
| Virus | 23 | 12 | 279 | 65 | 304 | 72 |
| Plant | 1 | 1 | 13 | 10 | 14 | 11 |
| others | 158 | 28 | 402 | 102 | 560 | 123 |
